# Supplementary material for: Optimizing Crop Production with Bacterial Inputs: Insights into Chemical Dialogue between Sphingomonas sediminicola and Pisum sativum
Source: Microorganisms. 2023 Jul 21;11(7):1847. doi: 10.3390/microorganisms11071847 (PMC10385058; doi:10.3390/microorganisms11071847)
Supplement: Supplementary file 1 [file microorganisms-11-01847-s001.zip › Figure S2.pdf]

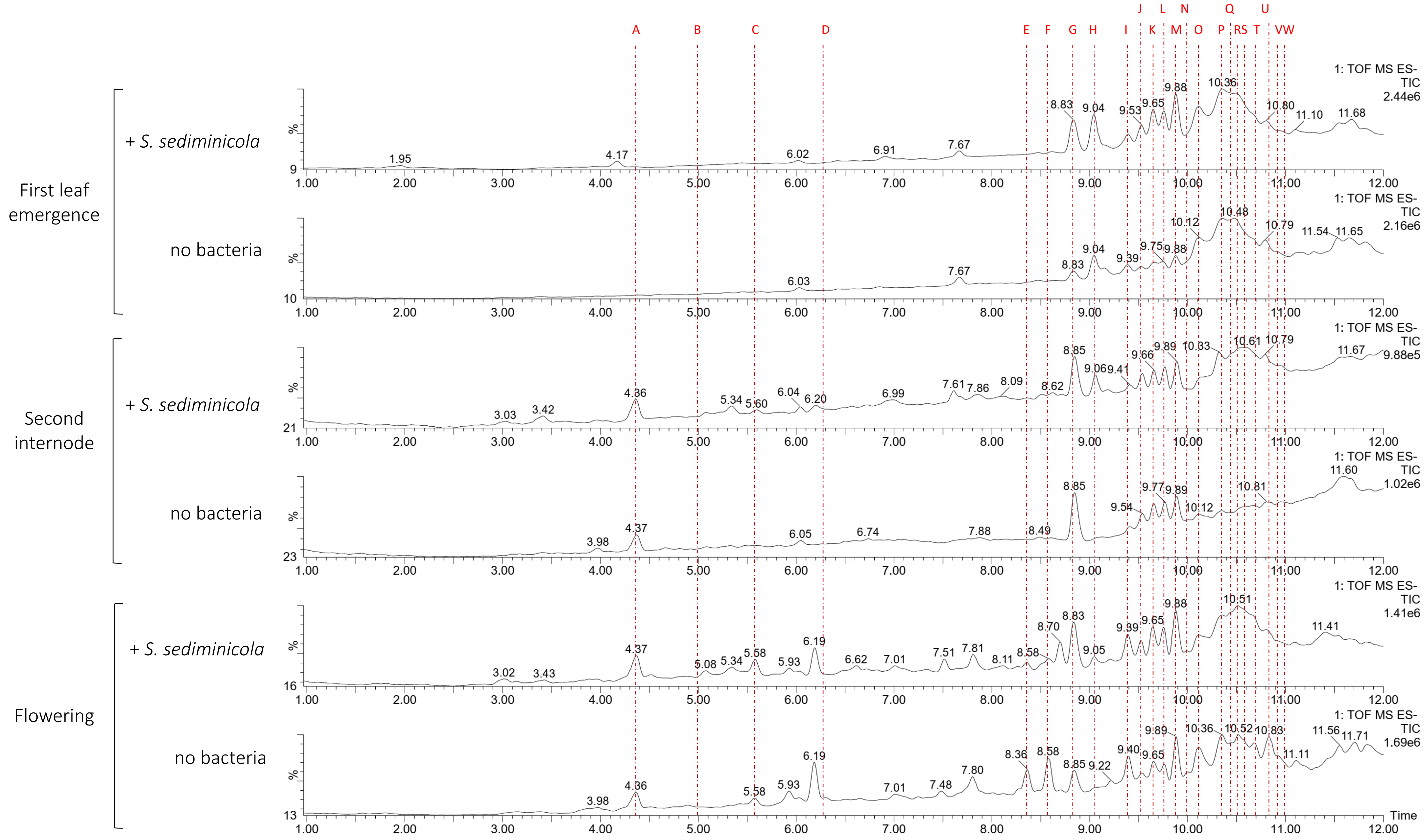

First leaf emergence
+ S. sediminicola
no bacteria
Second internode
+ S. sediminicola
no bacteria
Flowering
+ S. sediminicola
no bacteria
A
B
C
D
E
F
G
H
I
J
K
L
M
N
O
P
Q
RS
T
U
VW
1: TOF MS ES-
TIC
2.44e6
2.16e6
9.88e5
1.02e6
1.41e6
1.69e6
%
Time
