# Supplementary figures and images for: Optimizing Crop Production with Bacterial Inputs: Insights into Chemical Dialogue between Sphingomonas sediminicola and Pisum sativum
Source: Microorganisms. 2023 Jul 21;11(7):1847. doi: 10.3390/microorganisms11071847 (PMC10385058; doi:10.3390/microorganisms11071847)

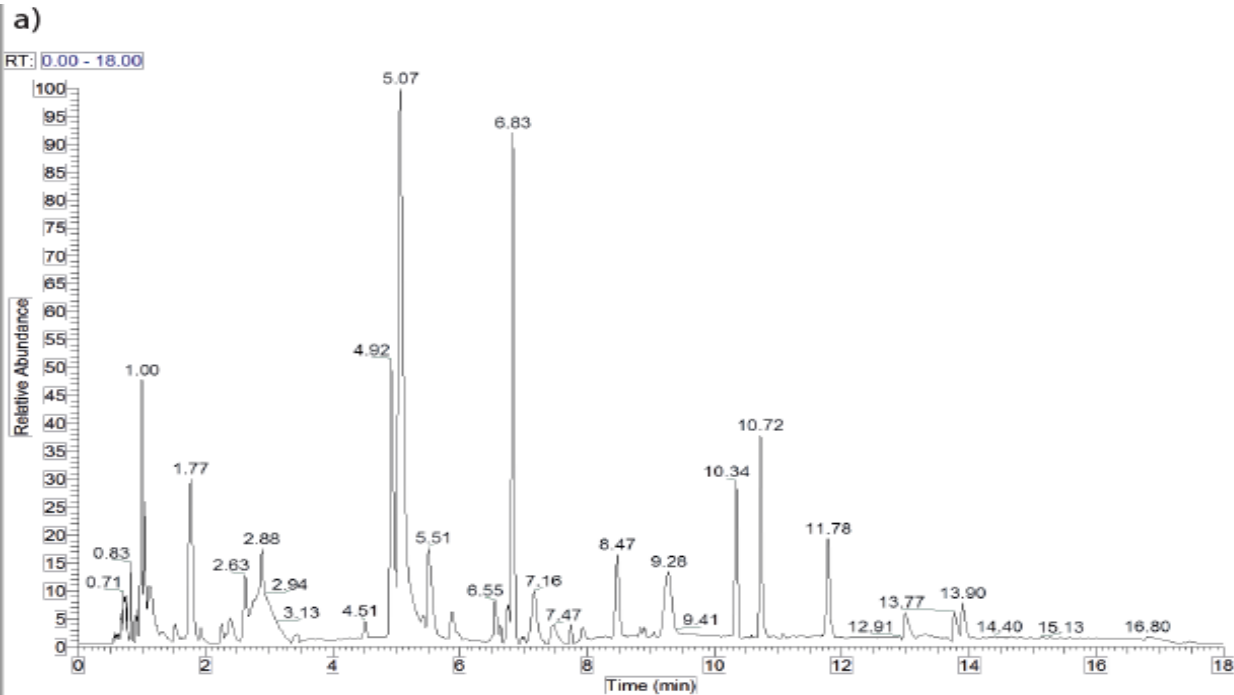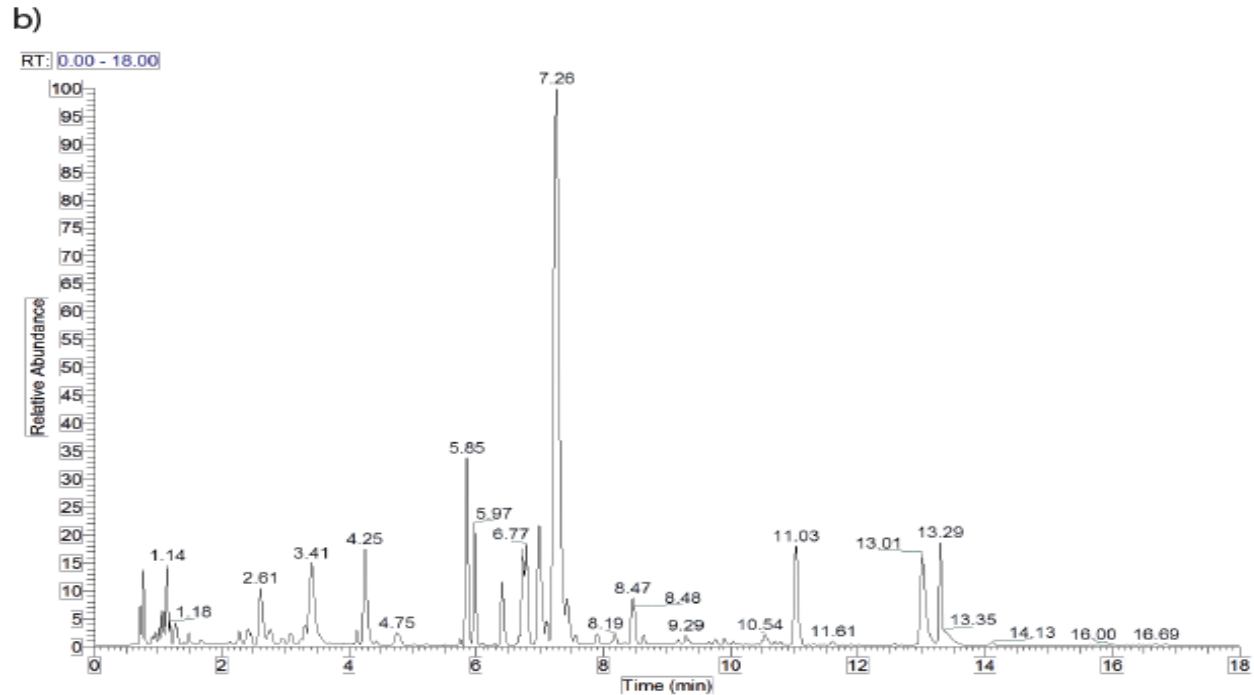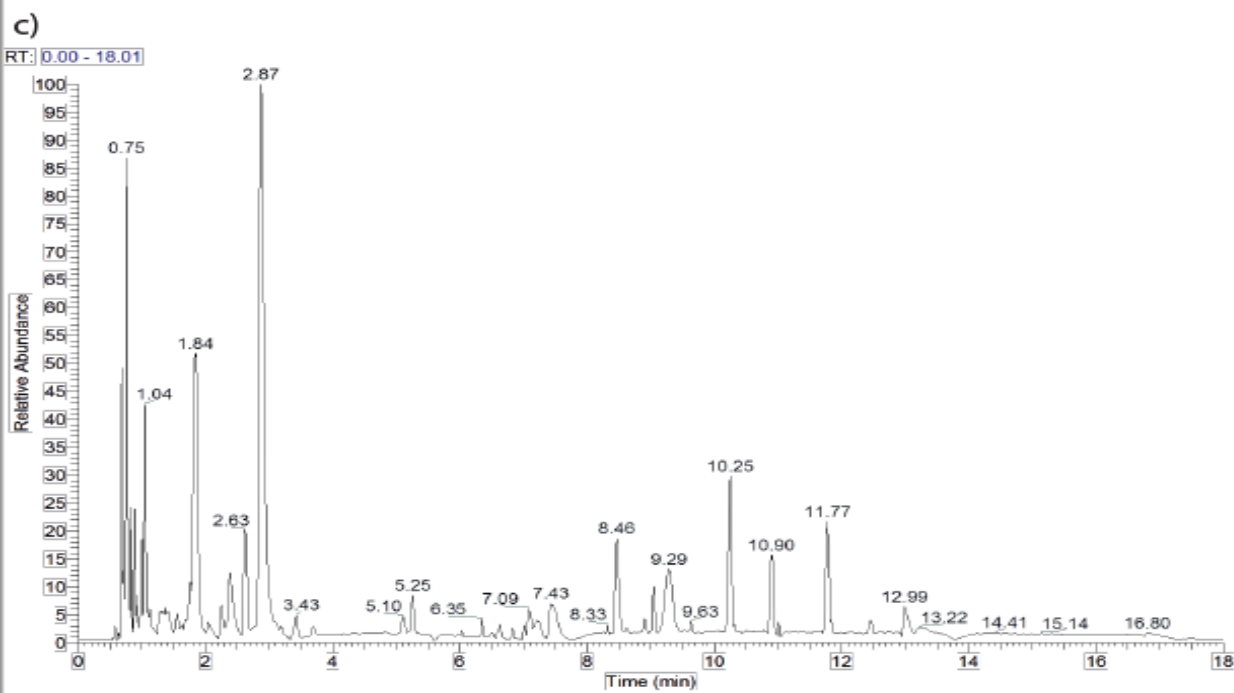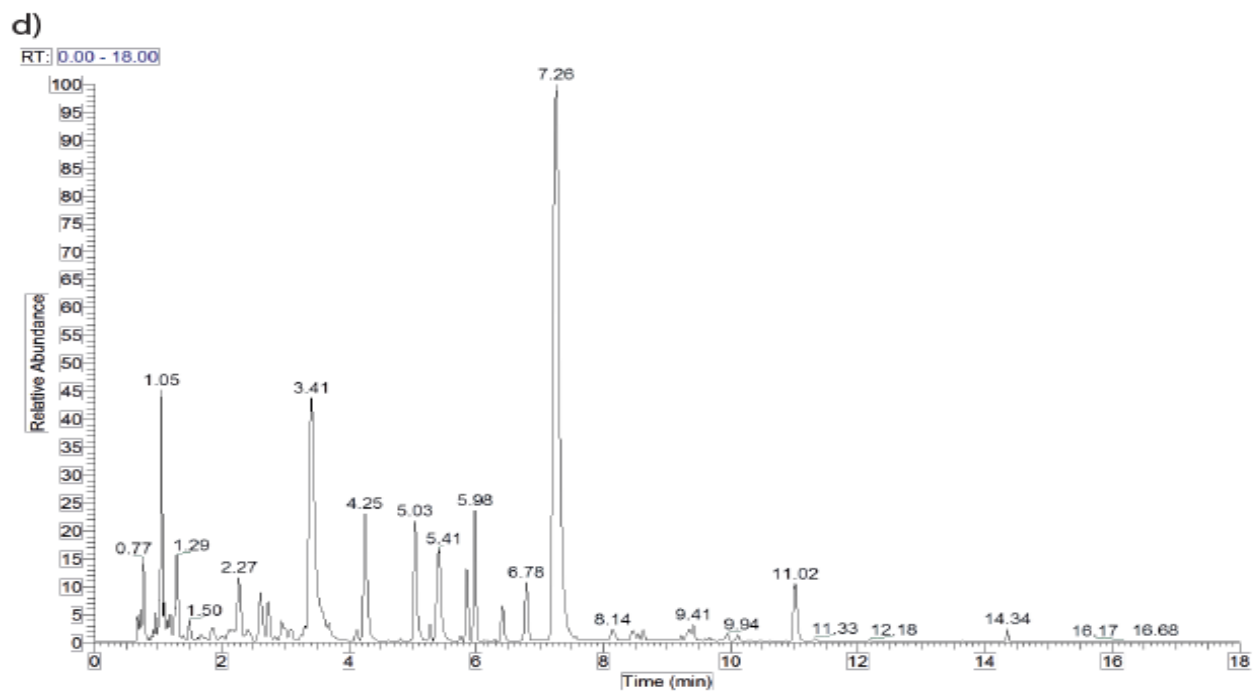

Supplement: Supplementary file 1 [file microorganisms-11-01847-s001.zip › Figure S1.pdf]
